# Revisiting Candidacy: What Might It Offer Cancer Prevention?

**DOI:** 10.3390/ijerph181910157

**Published:** 2021-09-27

**Authors:** Samantha Batchelor, Emma R. Miller, Belinda Lunnay, Sara Macdonald, Paul R. Ward

**Affiliations:** 1 Discipline of Public Health, Flinders University, Adelaide 5001, Australia; stur0028@flinders.edu.au (S.B.); emma.miller@flinders.edu.au (E.R.M.); belinda.lunnay@flinders.edu.au (B.L.); 2 General Practice and Primary Care, Institute of Health and Wellbeing, University of Glasgow, Glasgow G12 8QQ, UK; Sara.Macdonald@glasgow.ac.uk

**Keywords:** candidacy, lay epidemiology, breast cancer, cancer, modifiable risk factors, primary prevention

## Abstract

The notion of candidacy emerged three decades ago through Davison and colleagues’ exploration of people’s understanding of the causes of coronary heart disease. Candidacy was a mechanism to estimate one’s own or others risk of disease informed by their lay epidemiology. It could predict who would develop illness or explain why someone succumbed to it. Candidacy’s predictive ability, however, was fallible, and it was from this perspective that the public’s reticence to adhere to prevention messages could be explained, as ultimately anybody could be ‘at-risk’. This work continues to resonate in health research, with over 700 citations of Davison’s Candidacy paper. Less explored however, is the candidacy framework in its entirety in other illness spheres, where prevention efforts could potentially impact health outcomes. This paper revisits the candidacy framework to reconsider it use within prevention. In doing so, candidacy within coronary heart disease, suicide prevention, diabetes, and cancer will be examined, and key components of candidacy and how people negotiate their candidacy within differing disease contexts will be uncovered. The applicability of candidacy to address modifiable breast cancer risk factors or cancer prevention more broadly will be considered, as will the implications for public health policy.

## 1. Introduction

Cancer is a leading cause of death in Australia and around the world [1,2] and, despite steady improvements in cancer survival over recent decades, cancer incidence continues to rise [3,4]. Associated with this are cancer control efforts that target both primary and secondary prevention. 

Appropriate and effective action on primary prevention strategies is required to address cancer incidence, given that the World Health Organization estimates that 30–50% of cancers could be prevented through addressing behavioural, infectious and environmental risk factors [1]. Behavioural risk factors include smoking tobacco, alcohol consumption, and being overweight and obese. Current data suggest that tobacco and obesity are the leading modifiable risk factors for cancer [5,6]. Alcohol, in particular, is also causally linked to cancer and in 1998 the International Agency for Research on Cancer classified alcohol as a Group 1 carcinogen (known to cause cancer) [7]. Epidemiological evidence demonstrates an increased risk of cancer of the oropharynx, larynx, oesophagus, liver, colon, rectum and female breast resulting from consuming alcohol, with alcohol-attributable cancers at these sites comprising 5.8% of all cancer deaths world-wide [8]. Of current concern is the dose–response relationship between alcohol and increased breast cancer risk among women [9,10,11,12], as breast cancer is the most common cause of cancer mortality among women. In Australia, ‘mid-life’ women (aged between 45 and 64 years) are among the ‘heaviest’ drinkers, consuming alcohol at levels that pose harm more than in any other female age group [13]. Nonetheless, research shows that mid-life women have limited awareness of this link [10]. Without this understanding women are unlikely to consider the need to modify their consumption as a way to decrease their breast cancer risk. Therefore, there is a crucial need for appropriate public health strategies to address alcohol as a modifiable risk factor for breast cancer. 

Secondary prevention strategies, such as screening, have been a pillar of cancer control, contributing to increasing early detection of cancer and resulting in positive impacts on survival rates [3,14]. Whist some research suggests the public generally understand that screening is ‘almost always a good idea’ [15] (p. 64) given it can detect cancer at early stages, other research suggests a lack of knowledge regarding screening [16,17]. Issues such as service access, trust, and fear of screening and specifically, fear of the outcome of screening (i.e., a cancer diagnosis) deter people from participation in screening [17,18,19]. This is reflected in Australian data where relatively low screening coverage of the eligible population has been estimated: 37% for bowel cancer screening; 47% for cervical screening and 56% for breast cancer screening [20].

In contrast, research by Meyer et al. [10] suggests that an increased emphasis on the benefits of breast cancer screening may have led some Australian women to consider screening as a primary prevention approach, and thus they are less inclined to take action to address other modifiable risk factors such as decreasing alcohol consumption, maintaining a healthy weight and participating in physical activity. Pink ribbon culture that dominates the public consciousness regarding breast cancer may also impact primary prevention efforts. Pink ribbon culture has been described as ‘the movement of breast cancer from ‘ a stigmatised disease’ to ‘a cause’ around which people have formed a collective purpose of raising awareness and support’ [21] (p. 522). Within this culture there is a predominant emphasis on the importance of early detection through breast self-examination and screening, and on women’s empowerment and survivorship [21], and perhaps less emphasis on modifiable risk factors.

Further impediment to primary prevention is a lack of awareness of, or confusion about what are the modifiable risk factors for cancer. While research demonstrates that people readily identify modifiable cancer risk factors made more obvious by environmental and physical changes, such as tobacco and asbestos with lung disease and sun exposure with skin cancer, other modifiable risk factors such as consuming alcohol are less easily recognised [22].

In a survey of Australian adults, people reported feeling uncertainty about the link between alcohol and cancer; and 23% of those reporting they knew about the link, reported a belief that red wine had no negative effect on cancer risk, with 25% reporting they thought red wine decreased cancer risk [23]. Contributing to this confusion is mixed messages about whether alcohol is harmful or helpful to health, which have been promulgated by media commentary [24]. To clarify risks regarding alcohol, the Australian National Health and Medical Research Council guidelines were recently updated with the statement ‘there is no safe level of alcohol consumption’ [25]. Other research that examined people’s views of the link between alcohol and cancer showed participants were defensive or dismissive of cancer prevention messages [26] and were reluctant to consider behaviour change strategies given a prevailing perception that ‘everything causes cancer’ [27]. Whilst this aligns with previous research reflecting confusion about modifiable risk factors for cancer, it is also likely to reflect the high social acceptability and normalisation of alcohol consumption within society [28].

Public confusion about risk factors, is also apparent when considering breast cancer specifically. A study of women with no previous breast cancer diagnosis found the majority of women believed that breast cancer was related only to genetic or familial factors [29]. Whilst family history is indeed a risk factor, inherited cancer risk factors account for only approximately 5–10% of all breast cancer cases [30]. Of concern here is that such misconceptions have potential to negatively impact on women’s participation in both primary and secondary prevention behaviours [29,31], given their explanatory models of what causes cancer (i.e., it is familial or inherited), may lead them to incorrectly identify themselves as not at risk.

Given the discrepancies between lay understandings and scientific evidence [29,32,33], where many people hold the belief that cancer (except the link between smoking and cancer) cannot be prevented [34,35] and that ‘everything causes cancer’ [36], the challenge for primary cancer prevention is to increase awareness of modifiable risk factors and to address people’s resistance to incorporate these risks into their explanatory models of what contributes to cancer. Developing effective primary prevention strategies that address modifiable risk factors (both for cancer more broadly, and breast cancer specifically), therefore, first requires an improved understanding of people’s lay perceptions and beliefs about cancer, what informs these beliefs and how people apply their own knowledge of cancer risk factors to determine their own level of risk.

Candidacy, an individual’s estimation of disease risk, informed by their ‘lay epidemiology’ [37] may offer a novel approach to elucidate how the public perceive cancer risks, and how they respond to and act on this risk. What could an understanding of whether or not people consider themselves as cancer candidates and for what types of cancer contribute to the evidence regarding primary cancer prevention strategies?

Three decades ago the seminal work of Davison et al. [37] introduced this concept of candidacy in the context of coronary heart disease (CHD). Up to 2001 the work of Davison et al. [37] was the second most cited paper in ‘Sociology of Health and Illness’ [38] and to date has over 700 citations across extensive health research including: risk factors such as smoking [39,40], alcohol [41,42], obesity [43,44], issues such as injecting drug use [45], suicide prevention [46,47], primary health care [48], vaccination [49,50], and the exploration of women’s lived experience during the COVID-19 pandemic, what the authors termed ‘COVID-19 candidacy’ [51] and their alcohol consumption during this time [11], among others. When traversing these citations, it is apparent that concepts articulated within the candidacy framework continue to resonate in health research. Nevertheless, ‘candidacy’ as an interpretive framework to explore understandings of disease risk and how to tailor prevention efforts within other major illnesses or disease, including primary cancer prevention has received less attention.

The aim of this paper is therefore to present the key elements of the candidacy framework, and its application to date within CHD, suicide prevention, diabetes, and cancer. In doing so we will advance our understanding of candidacy with the purpose of cultivating ideas of what the candidacy system might mean for future cancer prevention research and its potential for public health interventions more generally. With regard to breast cancer, understanding what informs women’s lay perceptions of breast cancer risk and how these shape their acknowledgement (or not) of candidacy are important considerations to understand women’s willingness to address modifiable risk factors, such as alcohol consumption to reduce their breast cancer risk. To date breast cancer candidacy has not been explored from this perspective. Reconsidering the novel approach of candidacy in the context of primary cancer prevention may offer new understandings that can inform public health policy to increase women’s knowledge of and action toward reducing modifiable risk factors at individual levels that have population/ community level impact on cancer reduction.

## 2. How Risk Theory Informs Ideas of ‘Candidacy’

Given a central tenet of candidacy is an assessment of disease risk, being cognisant of the broader risk literature and the differing perspectives on risk can and should be explored. Taylor-Gooby and Zinn [52] provide a two-dimensional model mapping psychological and sociological approaches to risk along a continuum from realist to constructionist perspectives. This model aligns with Slovic’s work which argues for a multi-faceted approach to understanding risk [53,54] and, similarly, many other contemporary risk theorists argue that research should examine the links between psychological and sociological perspectives of risk. The mutual benefits of each approach can add value to the other and offer the prospect of cross-disciplinary research to enhance our understanding of lay perceptions of cancer risk more fully [52,53,55,56,57,58,59].

With particular reference to understanding ‘cancer candidacy’, Zinn [60] has called for research that attempts to understand the ways in which risk plays out within people’s everyday lives. Of significance here is the sociological risk literature that positions risk as a normal everyday experience, making its way into everyone’s lives within what Bauman calls ‘liquid modernity’ [61]. Moreover, in terms of cancer, the perceived randomness of cancer suggests we are all ‘at risk’ [62] (almost like Beck’s notion of the democratization of risk) [63].

We live in a world where risk is knitted into the fabric of our existence. Information about risk is everywhere—and is readily available to shape our perceptions of risk (at the click of a mouse or the press of a touchscreen on a smart phone). In the contemporary neo-liberal society within which individuals are urged to be self-governing and show responsibility for their health and outcomes, and alongside an almost endless amount of information on risks, there is the potential to cause ‘existential anxiety’ [64] or even a ‘culture of fear’ [65], whereby people are theorised to become quasi-researchers in the quest for expertise about their bodies [66]. Boundless and often contradictory (and sometimes even false) information on risks has the potential for all individuals within a risk society to see themselves as an expert, with the lines between expert and lay views blurring [67,68].

Therefore, a key question is how should public health interventions position cancer risk that will resonate with the population in a meaningful way? This is particularly important for alcohol as a modifiable cancer risk, given the high global consumption of alcohol, where even low levels of consumption are linked to cancer risk, especially in the case of breast cancer [69]. The imperative for public health remains to address modifiable risk factors at a population level to impact cancer incidence, whilst the challenge is to understand how this risk information resonates at an individual level. Candidacy is offered as a mechanism that may add value in exploring these risks, and to understand how individuals interpret them in the context of their own cancer candidacy.

## 3. The Emergence of Candidacy

Davison’s notion of candidacy [37] emerged through exploring lay understandings of CHD. In-depth interviews with 180 men and women from South Wales revealed that individual’s lay understandings of the causes of CHD were critical in shaping their own perceptions of disease risk and response to it. ‘Candidacy’ was based on numerous ’inputs,’ or sources of information: knowledge was gleaned in the public domain via health education, news, and media, and in one’s own personal sphere from observations about how illness occurred for others and affected themselves, family, friends, and those in their broader community. The term ‘lay epidemiology’ [37] was conceived to describe the consolidation of these inputs and the views and explanations individuals held regarding the occurrence and patterns of illness. Although much of the information people gained about CHD risks were at a population level, they were subsequently ascribed at an individual level—that is, to people they knew to explain patterns of illness. Through lay epidemiology, perceptions of health risk could be determined, candidacy for CHD assigned, and people’s logic for participation or non-participation in illness prevention behaviours could be explained.

Candidacy emerged as an explanatory framework for CHD that could predict the risk of illness or death in others from CHD or assess one’s own risk, and retrospectively explain other people’s illness/death from CHD, or one’s own illness. A ‘type’ of person was identified as a CHD candidate based on their physical appearance (e.g., being overweight, red-faced, or short of breath), their social context (e.g., family history, where a person lives or works) or their personal characteristics (e.g., smoking, alcohol consumption, being stressed). Through the process of recognising who is a disease ‘candidate’, people acknowledge the changes they must enact to prevent illness.

While candidacy offered these explanations of the type of person who could be a coronary candidate, the participants in Davison’s study acknowledged that the candidacy system was fallible. They identified exceptions to the rule about who ought to be a CHD candidate whereby individuals that met candidacy criteria never succumbed to disease, such as the ‘Uncle Norman’ figure who drinks and smokes all his life but lives to an old age—deemed by Davison et al. to be an ‘unwarranted survivor’ [70] (p. 682). Juxtaposed with this was an ‘Aunt Julia’ hypothetical figure—a slim and health conscious person [71] who somehow suddenly and unexpectedly suffers a heart attack; as described by Davison’s participants ‘the last person you’d expect’ and as such deemed by Davison et al. to be an ‘anomalous death’ [37] (p. 14).

Recognising these candidacy violations also provides a mechanism to refute risk [72]. Accounts by smokers who rationalise why smoking is not harmful to them reflect this [40], as does research exploring alcohol related harm: ‘he’d probably drink a bottle of vodka a day... and you think how’s this guy doin’ what he’s doin’ with no health [effects]’ [26] (p. 5). Furthermore, other mechanisms to mitigate, avert or distance oneself from risk such as risk comparison, denial, or techniques of risk neutralisation [40,45] may be used to deny disease candidacy.

Additionally, it is worth mentioning in this candidacy discussion, the occurrence and experience of serious illness, which has been recognised previously as ‘disruptive moments’ [73] or ‘biographical disruption’ [74]. Individuals may question ‘why me? why now?’ and in seeking answers to justify these disruptions, draw on explanatory models such as fatalism to explain why these events occur [71] (p. 44). Similarly, Davison’s participants employed notions of fate and chance saying ‘it can happen to anyone’, with Davison providing the analogy of pleasure boats to describe this, where at any time your boat could be called in ‘come in number 23, your time is up’ [70] (p. 681).

The inference being that regardless of whether you participate in a healthy lifestyle or not, ‘when your time is up, your time is up’. This contributes to public distrust of the certitude of public health messages which infer that addressing modifiable risk factors will prevent disease, and subsequently the dismissal of these messages impacts on individual’s willingness to participate in health promoting behaviours, due the improbability that they would have any effect [41,72]. Moreover, the fallibility of the candidacy system allows for the negation of risk as, according to these explanatory models, illness could strike regardless of whether or not you address risks [72].

## 4. Considering Lay Epidemiology as Key to Understanding Candidacy

Through Davison’s study, candidacy emerged as a way to understand lay epidemiology ‘the routine observation of cases of illness and death in personal networks and the public arena’ [70] (p. 678). Davison used this term in recognition of the similarity between lay and traditional epidemiology, in that both gather risk information at the population level and observe patterns of illness, but neither can predict exactly who will succumb to illness. Contrasting this, are several points of divergence, which we will now explore.

How lay epidemiology frames an issue differs to traditional epidemiology. Lay epidemiology often contradicts expert views, and a broad literature has examined this lay-expert divide—where scientific knowledge is privileged over lay knowledge [75,76,77]. Lay knowledge is experiential, contextual and observational, recognising issues at an individual or local level that scientific knowledge may not recognise or contest [77]. Many researchers argue that valuing and recognising lay knowledge as equal but different to expert knowledge is necessary for effective public health action [77,78,79].

Brown’s research [80] exploring community action regarding the apparent link between toxic waste and leukemia, demonstrates how lay knowledge (in this context termed ‘community epidemiology’), can bring to the attention of expert’s health issues or risks that may not otherwise have been seen or actioned. In this instance, ‘community epidemiology’ resulted in positive outcomes. Ignoring or contesting lay knowledge, however, can result in dire consequences as shown by the Grenfell Tower fire in London in 2017. Residents of the tower (lay people) had frequently raised concerns to authorities (the experts) concerning the management of fire hazards, only to have these concerns quashed. The disregard of the identified risks by lay people and lack of action by experts to address them resulted in a fire that caused the deaths of 72 people and likely trauma to many more [81].

Other research highlights these often-divergent views of lay epidemiology and science and the detriment of not including the lay views in the development of public health interventions [77,78]. One such example is an exploration of the lay epidemiology of people who inject drugs. The participants in this study were aware of the health risks associated with their drug use, but health outcomes for them were not their key priority, with harm minimisation strategies seemingly irrelevant against ‘everyday considerations such as drug supply, shelter and fear of incarceration’ [45] (p. 252). This is at odds with the health promotion strategies put in place that focus solely on the health outcomes. Similarly, an analysis of public views of alcohol drinking guidelines in the United Kingdom [42] identified a disconnect between the guidelines, framed exclusively from an epidemiological perspective regarding safe consumption for health outcomes, and people’s lay epidemiology that informed their interpretation of the relevance of their guidelines in assessing their own consumption levels. Participant’s positioned alcohol consumption and ‘health risks’ in the wider context of other concerns in their lives such as ‘the perceived benefits of risky activities, and individual and societal norms, attitudes, and values’ [42] (p. 1916). Acknowledging and incorporating lay views and understandings into the development of public health interventions is therefore paramount to ensure that they resonate with the target audience.

Cognisant of the above, Russel and Kelly [82] go further in suggesting that lay epidemiology should be included when setting research and policy agendas. Their review of correspondence from parents and grandparents of children with autism, was deemed ‘lay epidemiology in action’, describing a process where correspondents’ views were informed by ‘an interaction between personal, social, media, and scientific sources’ [82] (p. 129). It was apparent that, although not scientific, their lay evidence was largely credible and valid. This is consistent with research conducted by Wynne [76] and Lewis [83] who challenge the dichotomous lay-expert divide, proposing that both have a valid role to play in informing public health research and action on addressing health.

In the context of prevention efforts consideration of lay epidemiology is therefore crucial, and an exploration of candidacy brings another layer to this work. Women’s lay epidemiology regarding both alcohol and breast cancer may determine women’s assessment of their breast cancer candidacy. Their willingness to be involved in breast cancer prevention behaviours such as addressing modifiable lifestyle risk factors such as alcohol and screening may be contingent upon whether they see themselves as breast cancer candidates or not.

## 5. How Candidacy Has Been Applied in Other Contexts

Along with other research regarding CHD prevention [84,85,86], issues including suicide prevention [46,47], diabetes [87] and COVID-19 [51], have also been examined through a candidacy lens, offering extensions to the initial candidacy system devised by Davison, and these will now be considered.

One factor found to affect whether someone considers themselves or others to be a candidate is gender. A study by Emslie et al. [85] showed that almost all interviewees perceived males to be coronary candidates, despite CHD being the leading cause of death in women at the time. Moreover, Angus [86] demonstrates that women themselves resist their CHD candidacy, considering it as a ‘man’s problem’ and an interruption to their gendered caring roles. Nevertheless, some women did accept their candidacy and subsequently modified their behaviours to reduce disease risk (reversing candidacy). These changes, however, were fleeting, as they were impacted by the resistance of other family members to adopt healthy behaviours, making it necessary for women to renegotiate their risk in response to competing gendered roles and responsibilities.

Gender also affects candidacy in relation to suicide prevention and can impact individuals’ willingness to accept or act on signs of distress. This is particularly apparent in young men, where signs of distress are typically dismissed as ‘alcohol talk’ [47], and for young men who do express suicidal ideation whilst drunk, their concerns are quickly concealed or revoked once sober to reinstate ‘the hard man act’ to conform with gender norms [46] (p. 154) and consequently candidacy is denied.

The cultural familiarity of who presents as a ‘typical’ candidate also impacts willingness to assign candidacy to oneself or others. In suicide prevention studies often the deceased may be viewed as not the ‘suicidal type’ [47] (p. 3) where the reference points for ‘suicide candidacy’ do not match with someone who was ‘generally perceived to be a sociable person with a good life and no indication of mental health problems’ [46] (p. 156). Similarly, research regarding people recently diagnosed with diabetes showed some individuals viewed themselves as an anomalous case, as their apparent healthy lifestyle did not fit with their understandings of disease causation [88]. Both of these examples provide evidence of the divergence between lay and expert views and the biomedical and lay understandings of disease causation [75,77,79]. In contrast however, other research suggests the ability to objectively identify culturally familiar diabetic candidates (like coronary candidates) through personal characteristics and lifestyle factors is much more explicit, as shown by this interviewee, ‘Well I reckon that if you’re over 50, overweight, and have hypertension, you’re a dead ringer for it! It’s just the classic symptoms’ [89] (p. 2375).

Resisted candidacy is another way candidacy may unfold. As described earlier, for women, coronary candidacy may be resisted based on gender, but candidacy can also be resisted based on the denial of risk factors. For example, individuals at-risk for diabetes within one study, acknowledged their risk factors, but denied these increased their candidacy and subsequently declined pre-diabetic screening, effectively resisting their candidacy [90]. Similarly, individuals who downplay their genetic or family history and instead highlight their healthy lifestyle behaviours are attempting to resist or re-shape their candidacy, ‘I wouldn’t have thought I would be a candidate. I’m a healthy eater and we walk a lot. I’ve always exercised’ [87] (p. 43). In this way, individuals’ distance themselves from the stereotype of a diabetic candidate (unhealthy or obese) and are able to exclude their own candidacy based on this.

Competing candidacy is also apparent, where other issues override the ability to accept or focus on candidacy. The need for individuals to prioritise other roles and responsibilities, prioritise other health issues or choose not to change associated lifestyle behaviours (should they accept their candidacy) [87], all provide examples of reasons for competing candidacy.

## 6. Exploring Cancer Candidacy

A large body of literature has explored lay understandings of cancer, however research specifically examining cancer candidacy is more limited. Two systematic qualitative reviews have captured the breadth of work regarding lay understandings of cancer. These reviews demonstrate how lay epidemiology shapes cancer related beliefs and behaviours, and the personal experience of cancer is instrumental in forming these views: ‘Witnessing the treatment, and sometimes death, of someone close was perceived as the most reliable source of information’ [91] (p. 1697). When considering risk, techniques such as suppressing and simplifying information, making comparisons, and rationalising beliefs acted to modify risk perceptions [91,92] and the existence of anomalous cases/deaths confused the view of who ‘gets’ cancer. Nevertheless, there remained a sense for some that cancer could be averted by avoiding unhealthy lifestyles [91].

Through 31 in-depth interviews Macdonald et al. [62] sought to explore the ‘ordinary views’ of people without a cancer diagnosis to understand their lay epidemiology and whether a particular ‘type of person’ could be identified as a cancer candidate. Consistent with previous research, these interviewees also formed their evidence base on ‘varied sources ranging from close personal and family experience, information gleaned from social networks, mainstream health education messages and media representations’ (p. 580). However, unlike the CHD studies, these interviewees found it more difficult to identify cancer candidates. Explaining this, the authors suggest that coronary candidates are more culturally familiar than cancer candidates, and that cancer risk factors are not as well understood. The only clear candidates identified were people who smoked—candidates for lung cancer; and sun worshippers—candidates for skin cancer, where interviewees could see a clear link between cause and effect. Cancer fear was also noted as a reason that precluded interviewees from identifying themselves as candidates or placing that burden on others; and in this way candidacy was resisted. In a later study examining individuals’ appraisals of their symptoms post their diagnosis, Macdonald [93] highlights that both shared and public narratives of cancer impact candidacy, with the theme of cancer being unpredictable, random, and down to luck dominating the discourse and thus negating the need to assign candidacy.

Two studies conducted in the United States (US) explored breast cancer candidacy. The first of these examined candidacy in relation to mammography screening through focus groups with women from diverse cultural backgrounds [94]. Candidacy for these women arose with less emphasis on lifestyle issues (as for coronary candidates), but with greater emphasis on reproductive histories, with women identifying those who had never had children or breastfed as potential candidates. Ethnicity also appeared as a considered characteristic, as women of different ethnicities compared their risk against others. A link between candidacy and screening was not well established, with a clear connection recognised only for women with a family history of breast cancer. Whilst candidacy emerged as a concept, anomalous deaths and unwarranted survivors did not, which the authors proposed might be due to women’s limited understandings of breast cancer causes. Suggested by this work, however, is the notion that candidacy, like ethnicity, is culturally constructed [94].

The second study exploring breast cancer candidacy conducted focus groups with women from African American neighbourhoods in the US [95]. These women utilised their shared memories and experiences (termed collective memory) to articulate breast cancer risk. While the women provided descriptors of breast cancer candidates, attributing this candidacy to themselves or others was limited. Explaining, this the authors suggest that ‘individual concern about breast cancer pales in comparison to the competing risks of daily survival, particularly in light of multiple responsibilities related to caring for and supporting their families’ [95] (p. 608). Thus, these data reinforce the impact of gendered roles and expectations on considerations of disease risk, providing another example of competing candidacy.

Numerous researchers have examined understandings of colorectal cancer and candidacy in the context of bowel cancer screening. Javanparast et al. [18] and Ward et al. [17] identified components of lay epidemiology that acted as barriers or enablers for screening; experiencing colorectal cancer in your close networks and having heard about the program facilitated an understanding of screening need, whilst those who held fatalistic views regarding cancer were less likely to see a need to attend screening. Bikker et al. [96] conducted 61 in-depth interviews with men and women who identified themselves as either colorectal screeners or non-screeners to more closely explore components of candidacy. Findings were consistent with Macdonald et al. [62] in that cancer fear was commonly described; it was difficult to illustrate a type of person who was a candidate; and participants’ views of anomalous cases and risk perceptions were wide. There was a common belief that ‘anyone can be a candidate for cancer’ [96] (p. 360) and, again, participants were reluctant to assign candidacy to themselves or others, although they were more likely to see themselves as candidates for screening.

## 7. A Proposed Framework for the Components and Enactment of Candidacy

Through this review, key components of Davison’s original candidacy system have been presented (lay epidemiology, assessing risk, identifying anomalous cases and unwarranted deaths, and the culturally familiarity of candidates) as shown in Table 1. An analysis of candidacy in other health contexts has illustrated commonalities with Davison’s candidacy system, as well as the emergence of different components of candidacy and how it is enacted, as shown in Table 2.

The key components of Davison’s candidacy system were seen across CHD, diabetes, suicide prevention and cancer. Within CHD and diabetes candidates were culturally familiar, conjuring images of the type of person that may be a candidate for illness, for example the ‘dead ringer’ for a diabetic candidate [89] (p. 2375). This comes with both benefits and burdens. Whilst a culturally familiar stereotype might be useful to highlight risk factors, people also may resist these and deny their candidacy. For example, downplaying genetic risk and risk behaviours while highlighting healthy ones is one way in which people can resist their candidacy.

Candidacy was also shown to be gendered and linked with gendered norms, roles, and responsibilities. This was highlighted within ‘suicidal candidacy’ of males and the resistance of CHD candidacy for females. Competing or contested candidacy was also apparent by means of one illness taking precedence over another (competing) or where social roles, identities or lifestyle issues take prominence over acknowledging one’s risk for illness (contested candidacy) or acting on addressing that risk. In some instances, however, individuals may have sufficient agency to address risk factors and enact lifestyle changes, and thus reverse candidacy. This is exemplified by the pre-diabetic individual who addresses lifestyle factors working to reduce or remove their diabetic risk and thus candidacy. In exploring suicide prevention, the literature suggests that widely held assumptions about what a suicidal person is like (depressed and withdrawn) can inadvertently result in overlooking those who are suicidal yet not presenting as such, termed ‘missed candidacy’. Lastly, candidacy can be understood as being collective, as illustrated in studies that have highlighted the social patterning of candidacy.

## 8. Candidacy for Cancer Prevention?

Whilst some exploration of the candidacy concept has occurred in relation to cancer, it is perhaps timely to revisit the potential usefulness of this, particularly for those cancers that may have modifiable risk factors. As identified by Macdonald [62] (p. 577) ‘for cancer candidacy work, cancer must be equally well understood and as culturally resonant as CHD.’ In the instance of breast cancer, the impact of campaigns such as pink ribbon and high-profile celebrity breast cancer cases may well have raised the consciousness among women of breast cancer and informed their lay epidemiology. How this translates to the assessment of their own or other’s candidacy for breast cancer, and how this impacts their participation in both primary prevention activities such as reducing alcohol, increasing physical activity, and addressing weight, as well as their participation in secondary prevention activities is yet to be understood. However, the implications of this for public health prevention efforts, would seem an important focus for future exploration.

## 9. Conclusions

The key purpose of this paper was to present the concept of candidacy, from its emergence within CHD to its application in other health contexts, and to elucidate the key components of the concept. In doing so, important questions regarding the applicability of the concept within primary cancer prevention have been posed. Re-purposing the interpretive framework of candidacy to further explore primary cancer prevention may be timely, given increasing scientific understandings of potentially modifiable cancer risk factors [1,97]. It is also a time when the public discourse of cancer as the most feared disease is slowly moving towards more positive perspectives [98], whereby treatment advances, willingness of cancer survivors to discuss their journey and media coverage of positive cancer outcomes work to alleviate the stigma associated with cancer [35]. These factors may be shifting the lay epidemiology of some sections of the public, their appraisal of risk and their likelihood of considering candidacy, thus opening up new opportunities to examine prevention efforts that better resonate with the community. Similarly, for breast cancer, ‘Pink’ campaigns that are synonymous with the disease, may be shifting women’s ‘lay epidemiology’; better equipping women to appraise risk factors and successfully identify their own or other’s candidacy, with participation in prevention activities potentially contingent on this. These shifts in lay epidemiology could mean that cancer candidacy functions in different ways: for different genders, different cancers and in different social contexts; but answers to these questions are yet to be examined.

Given that breast cancer remains the highest cause of cancer death in women, the clear epidemiological link between alcohol and breast cancer, and that mid-life women are high consumers of alcohol, it would seem a high proportion of women are indeed potential cancer candidates, notwithstanding other possible risk factors that may confirm their candidacy. As identified by Macdonald et al. [62], in considering candidacy ‘there is scope to develop the concept both to encourage cancer candidacy or challenge misconceptions about candidacy’ (p. 588) and in turn inform more appropriate, relevant primary prevention strategies. Moreover, the success of primary prevention efforts to address modifiable risk factors for cancer will have positive implications for prevention of other non-communicable diseases of which many share the same modifiable risks as cancer [14,99].

## Figures and Tables

**Table 1:** Components of Candidacy.

Candidacy Is:	
Evidence informed	Lay epidemiology—sources of information that people gather at a micro, meso and macro level inform their framework of understanding and interpretation
An assessment of disease risk	Determined by ones lay epidemiology. Incorporates both perceived and comparative risks and the evaluation and interpretation of these risks enables one to assign (or not) candidacy to oneself or others. Applies what is seen at the population level to an individual level.
Fallible	Anomalous deaths and unwarranted survivors are a clear outcome of applying what is seen at a population level to the individual level. Adds to the belief that succumbing to serious illness is based on just fate or luck.
Culturally familiar	Stereotypes are readily recognisable—those with certain personal characteristics are readily identified as candidates, e.g., the red-faced, overweight coronary candidate, the leather skinned, tanned skin cancer candidate, the heavy smoker who is a lung cancer candidate

**Table 2:** Enactment of Candidacy.

Candidacy Can Be:	
Gendered	The predominance of the male coronary candidate
Resisted	Resistance (in the face unmistakable evidence) to assigning the label to oneself or others Related to the fear and dread Risk factors may be present, but ‘offset’ with other healthy behavior, thus deeming that candidate label inaccurate.
Competing or contested	Competing or contested candidacies may be linked to competing roles or social identity, (particularly for women) or other illnesses.
Reversed	Re-negotiating or addressing risk could see candidacy reversed
Collective	Candidacy may be socially structured by place or group

## Data Availability

No new data were created or analyzed in this study. Data sharing is not applicable to this article.
